# Dynamic changes in marker components during the stir-frying of Pharbitidis Semen, and network analysis of its potential effects on nephritis

**DOI:** 10.3389/fphar.2023.1123476

**Published:** 2023-03-14

**Authors:** Yuman Li, Yuhe Lu, Yujie Zhu, Jingchun Yao, Haibing Hua, Jinyang Shen, Xun Gao, Kunming Qin

**Affiliations:** ^1^ Jiangsu Key Laboratory of Marine Bioresources and Environment, Jiangsu Ocean University, Lianyungang, China; ^2^ School of Pharmacy, Jiangsu Ocean University, Lianyungang, China; ^3^ Co-Innovation Center of Jiangsu Marine Bio-industry Technology, Jiangsu Ocean University, Lianyungang, China; ^4^ Lunan Pharmaceutical Group Limited by Share Ltd, Linyi, China; ^5^ Jiangyin Hospital Affiliated to Nanjing University of Chinese Medicine, Wuxi, China

**Keywords:** Pharbitidis Semen, stir-fried processing, network analysis, molecular docking, nephritis

## Abstract

**Introduction:** Pharbitidis Semen (PS) has been widely used in traditional Chinese medicine to treat several diseases such as nephritis. PS is usually stir-fried to enhance its therapeutic efficacy before use in clinical practice. However, the changes in phenolic acids during stir-frying and the mechanisms of their therapeutic effects on nephritis are still unclear.

**Methods:** Here, we studied the processing-induced chemical changes and elucidated the mechanism of PS in the treatment of nephritis. We determined the levels of the 7 phenolic acids in raw PS (RPS) and stir-fried PS (SPS) using high-performance liquid chromatography, analyzed the dynamic compositional changes during stir-frying, and used network analysis and molecular docking to predict and verify compound targets and pathways corresponding to nephritis.

**Results:** The dynamic changes in the 7 phenolic acids in PS during stir-frying are suggestive of a transesterification reaction. Pathway analysis revealed that the targets of nephritis were mainly enriched in the AGE-RAGE, hypoxia-inducible factor-1, interleukin-17, and tumor necrosis factor signaling pathways among others. Molecular docking results showed that the 7 phenolic acids had good binding ability with the key nephritic targets.

**Discussion:** The potential pharmaceutical basis, targets, and mechanisms of PS in treating nephritis were explored. Our findings provide a scientific basis for the clinical use of PS in treating nephritis.

## 1 Introduction

Pharbitidis Semen (PS), the dried seeds of *Pharbitis nil* L.) Choisy or *Pharbitis purpura* L.) Voigt, a plant of the Convolvulaceae family, is a well-known traditional Chinese medicine (TCM) (Liu et al., 2019). More than 211 chemical components of PS have been reported, including phenolic acids, terpenoids, resin glycosides, fatty acids, alkaloids, and volatile oils. These chemical components have significant pharmacological effects, such as increasing the propulsion rate of the large intestine, promoting gastrointestinal peristalsis, treating nephritis, and expelling parasites, as well as anticancer, phlegm-resolving, anti-inflammatory, and immunity-improving effects (Choi et al., 2015; Xiang et al., 2017). Traditionally, PS is processed by plain stir-baking in accordance with “Lei’s Treatise on the Processing of Drugs,” a guideline book on the processing of TCM to yield active medicines (Gao et al., 2022). The process involves placing clean PS in a pot and stir-baking it in a slow fire until the seeds turn slightly brown. However, to the best of our knowledge, the dynamic phytochemical changes occurring in PS during processing have not been investigated. It has been reported that phenolic acids may be the main marker ingredients, which are effective in the treatment of nephritis (Zhou et al., 2011). Specific phenolic acids such as neochlorogenic acid (5-CQA), chlorogenic acid (3-CQA), cryptochlorogenic acid (4-CQA), caffeic acid (CA), isochlorogenic acid B (3,4-DiCQA), isochlorogenic acid A (3,5-DiCQA), and isochlorogenic acid C (4,5-DiCQA), the contents of these phenolic acids change significantly during stir-frying (Li, 2016). However, the dynamic changes that these compounds undergo during stir-frying are unclear.

Nephritis is a common renal disorder and a leading cause of end-stage renal disease. Nephritis can present in protean ways, with general features including proteinuria, hematuria, renal failure, and hypertension. Recent advances in nephritis management indicate that early therapeutic intervention can lead to the improvement in renal function and the long-term preservation of renal function, or slow the progression to end-stage renal failure in many cases. Several studies have reported the advantages of TCM in the treatment of nephritis, among which PS is a commonly used TCM (Li, 2018). However, the key ingredients and mechanism of PS in treating nephritis are still unclear. In recent years, many researchers have conducted pharmacokinetic studies on the main phenolic acids in PS (Li et al., 2020; Yang et al., 2020) and found that the phenolic acids in PS can be absorbed into the systemic circulation and exert pharmacological effects (Li et al., 2021; Li et al., 2022). More importantly, previous studies have also reported the potential of phenolic acids such as CA and 3-CQA in treating nephritis (Sidhoum et al., 2020; Song et al., 2022).

**FIGURE 1:**
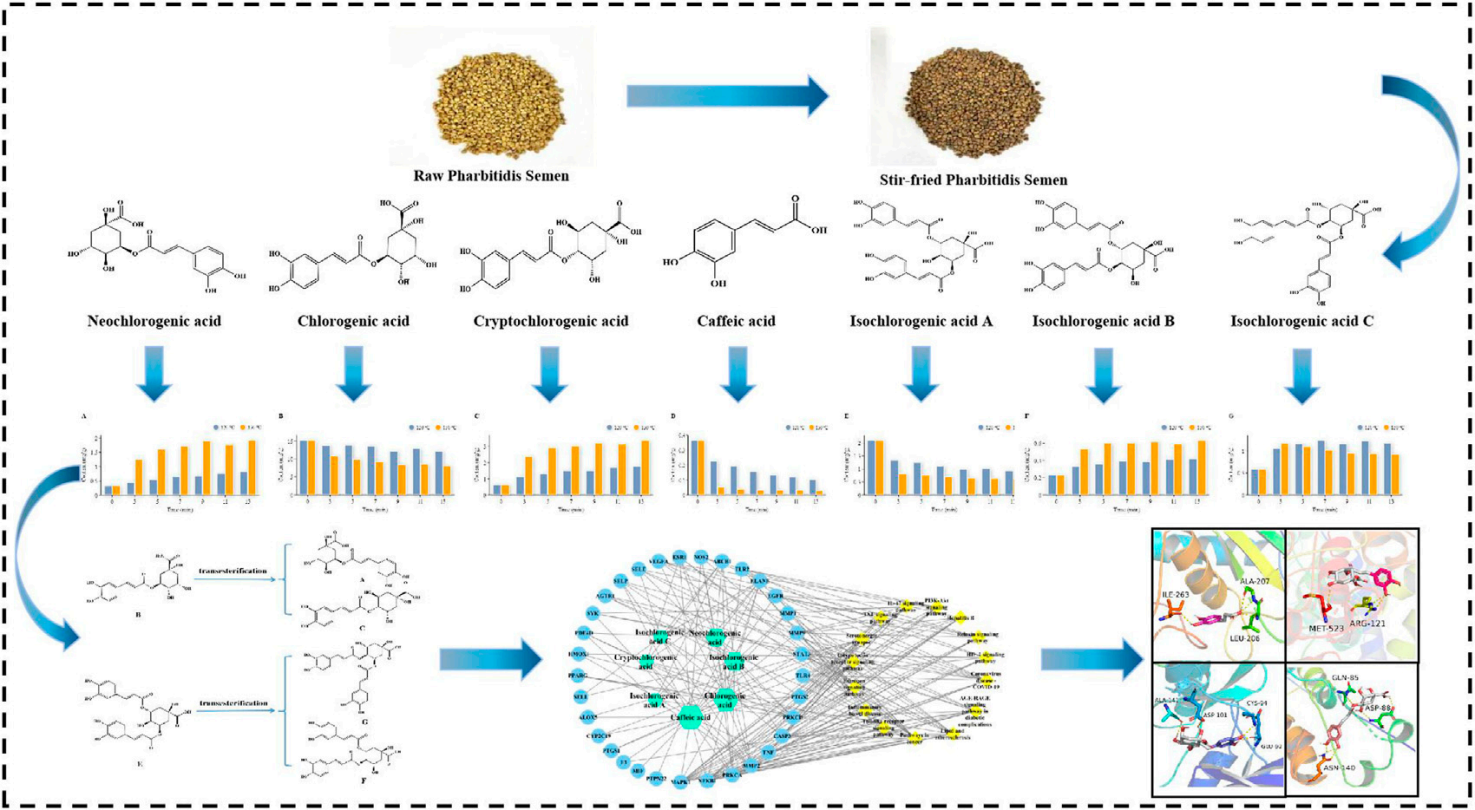
Flow chart of the study process.

Systems pharmacology is developed based on the cross-disciplines of directional pharmacology, systems biology, and mathematics, and has the characteristics of integrity and synergy in the treatment process using TCM. Therefore, it is suitable for analyzing the key ingredients and mechanisms of TCM in treating complex diseases. Network analysis is an analytical tool that integrates systems biology, multidirectional pharmacology, computational biology, and other emerging concepts and methods. It has been widely used for the functional analysis of drugs (Zhang et al., 2022). Here, we studied the differences in composition during the stir-frying of raw PS (RPS) and stir-fried PS (SPS) using high-performance liquid chromatography (HPLC) and explored their mechanisms of action in treating nephritis using network analysis and molecular docking. Our findings reveal the material basis and potential efficacy of PS in treating nephritis and provide a scientific basis for the clinical use of stir-fried PS in the treatment of nephritis as well as for the development of novel drugs to treat nephritis (Jin et al., 2021). A flowchart of the study process is shown in Figure 1.

## 2 Materials and methods

### 2.1 Reagents and materials

**FIGURE 2:**
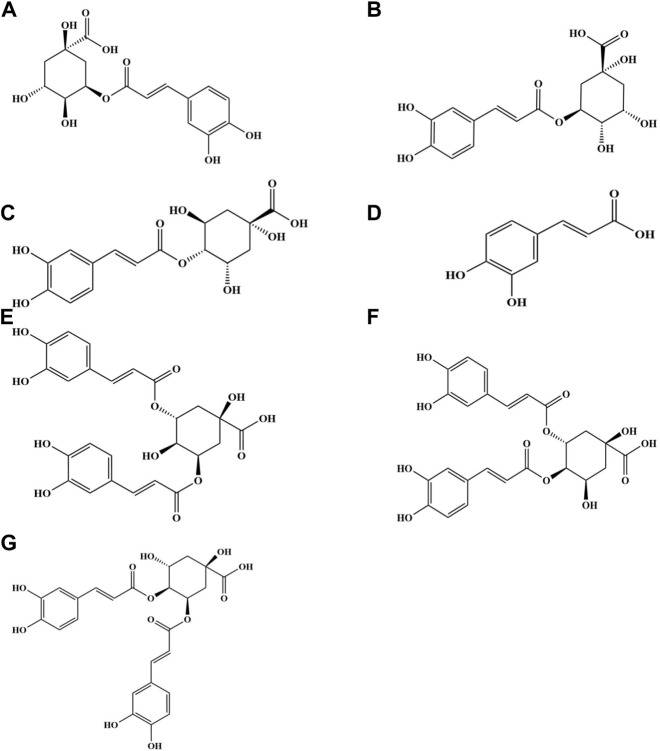
Chemical structure of the 7 phenolic acids in PS. **(A)**: 5-CQA; **(B)**: 3-CQA; **(C)**: 4-CQA; **(D)**: CA; **(E)**: 3,5-DiCQA; **(F)**: 3,4-DiCQA; **(G)**: 4,5-DiCQA.

All chemicals and reagents were of analytical grade, and the solvents used for chromatography were of HPLC grade. Methanol (MeOH) and formic acid were purchased from Shanghai Titan Technology Co., Ltd., Chromatographically pure MeOH was obtained from Sigma-Aldrich (Shanghai) Trading Co., Ltd., and purified water was acquired from Hangzhou Wahaha Group Co., Ltd. The standard chemicals included 3-CQA (batch: PS000627), CA (batch: PS010522), 5-CQA (batch: PS000974), 4-CQA (batch: PS001109), 3,5-DiCQA (batch: PS012051), 3,4-DiCQA (batch number: PS012052), and 4,5-DiCQA (batch number: PS001057), which were obtained from Chengdu Pusi Biotechnology Co., Ltd (Lukas et al., 2021). PS samples were purchased from 7 provinces in China in April 2022 and authenticated by Professor Kunming Qin, School of Pharmacy, Jiangsu Ocean University (Table 1). A voucher specimen is deposited at the School of Pharmacy, Jiangsu Ocean University. The structural formulae of the 7 phenolic acids in PS are shown in Figure 2.

**TABLE 1:** 14 batches of PS samples from different habitats.

Numbering	Origin	Batch number	Factory
Q1	Hebei province, China	2010002	Anguo juyaotang pharmaceutical Co., Ltd.,
Q2	Hebei province, China	2010003	Anguo juyaotang pharmaceutical Co., Ltd.,
Q3	Hebei province, China	200208	Anhui guanghe traditional chinese medicine Co., Ltd.,
Q4	Anhui province, China	201001	Bozhou jingwan chinese herbal pieces factory
Q5	Anhui province, China	210105	Bozhou zhongqiang chinese herbal pieces Co., Ltd.
Q6	Jiangsu province, China	181201	Anhui suntech chinese herbal pieces Co., Ltd.
Q7	Jiangsu province, China	181201	Anhui suntech chinese herbal pieces Co., Ltd.
Q8	Henan province, China	201210	Bozhou yonggang decoction piece factory Co., Ltd.
Q9	Henan province, China	200826	Bozhou yonggang decoction piece factory Co., Ltd.
Q10	Liaoning province, China	170614	Bozhou yonggang decoction piece factory Co., Ltd.
Q11	Liaoning province, China	171128	Bozhou yonggang decoction piece factory Co., Ltd.
Q12	Sichuan province, China	190601	Sichuan zhongxing pharmaceutical Co., Ltd.
Q13	Sichuan province, China	190601	Sichuan zhongxing pharmaceutical Co., Ltd.
Q14	Zhejiang province, China	20190301	Guangdong huiqun traditional chinese medicine Co., Ltd.,

### 2.2 Preparation of sample solutions

RPS and SPS samples were crushed in an FW-40 high-speed multifunction pulverizer mill (Shanghai Yiyan Test Equipment Co., Ltd.,). The dried sample (1.0 g) was accurately weighed and dispersed in 50 mL MeOH. After 30 min of ultrasonication, the sample was cooled to room temperature (in 25°C) and filtered. The filtrate was collected and transferred to a 25-mL volumetric flask, and the volume was made up with MeOH. Then, the sample was filtered through a 0.22-µm filter after thorough mixing to obtain the sample solution.

### 2.3 Chromatographic conditions

Samples were analyzed using an analytical SHIMADZU LC-20AD system (Shimadzu Corporation, Japan) equipped with a Kromasil 100-5-C18 column (4.6 × 250 mm, 5 μm) and an SPD-20A ultraviolet detector. The detection wavelength was 325 nm for the 7 phenolic acids. Analytes were eluted at a flow rate of 1 mL/min using a gradient profile at a column temperature of 25°C. The solvent system was composed of 0.1% aqueous formic acid solution A) and MeOH B). Gradient elution was performed as follows: 0–10 min: 10%–20% B), 20–30 min: 20%–30% B), 20–50 min: 30%–50% B), 50–51 min: 50%–10% B), 51–60 min: 10% B). Chromatographic data were processed using a Lab Solutions workstation (Bai et al., 2020).

### 2.4 Network construction

All chemical ingredients were obtained from the Traditional Chinese Medicine Systems Pharmacology Database and Analysis Platform (TCMSP, http://lsp.nwu.edu.cn/tcmsp.php). PubChem (https://pubchem.ncbi.nlm.nih.gov/) was used to obtain SDF files with 2D structures of the 7 components, namely 3-CQA, CA, 5-CQA, 4-CQA, 3,5-DiCQA, 3,4-DiCQA, and 4,5-DiCQA. SwissTargetPrediction is an online web-based tool established in 2014 to perform ligand-based target prediction for small bioactive molecules. The SwissTargetPrediction model was trained by fitting multiple logistic regression on various size-related subsets of known actives to weigh 2D and 3D similarity parameters in a so-called Combined-Score. A Combined-Score >0.5 predicts that the molecules are likely to share a common protein target. In reverse screening, the Combined-Score allows to calculate for any query molecule assumed as bioactive and determine the probability to target a given protein. As the 2D and 3D descriptions of molecules are complementary, this dual scoring ligand–based reverse screening showed high performance in predicting the macromolecular targets in various test sets (Daina et al., 2019). The canonical SMILES of the compounds were uploaded into the SwissTargetPrediction database (http://www.swisstargetprediction.ch/), the Herbal Ingredients’ Targets (HIT) Platform 2.0 (http://www.badd-cao.net:2345/), and the HERB Materia Medica Database (http://herb.ac.cn/) to obtain UniProt IDs for predicting targets with the organism *Homo sapiens* (Ou et al., 2020).

The biological targets related to nephritis were selected from the OMIM (https://omim.org/), TTD (https://idrblab.net/ttd/), and GeneCards (https://www.genecards.org/) databases using “nephritis, glomerulonephritis” as the keywords (Guo et al., 2019). Microsoft Excel was used to input the targets of the 7 phenolic acids and nephritis-associated targets in 2 columns, which were compared to identify the common targets that were potential targets (Xi et al., 2022). The intersection targets were imported into the STRING database (https://string-db.org/), “Multiple proteins” was selected, the species was set to “Homo Sapiens,” and the minimum interaction score was set to medium confidence (0.4) to obtain the protein–protein interaction network diagram. The resulting TSV file was imported into Cytoscape 3.7.1 for visual analysis of the network (Xia et al., 2020).

The Gene Ontology (GO) biological process (BP) was used to further validate that the potential targets were indeed matches for nephritis. GO and Kyoto Encyclopedia of Genes and Genomes (KEGG) signaling pathway analyses were performed using the Database for Annotation, and visualization (http://www.bioinformatics.com.cn/) was using *p* ≤ 0.01 (Lin et al., 2022). Compound–target, disease–target, and target–pathway networks were constructed using Cytoscape 3.7.1 (Bethesda, MD, USA). In these bilateral networks, the nodes represent the compounds, diseases, targets, or signaling pathways, whereas the edges represent their interactions (Cao et al., 2022).

### 2.5 Molecular docking

The 3D structures of phenolic acids were obtained using the PubChem database (https://pubchem.ncbi.nlm.nih.gov/) and saved in the SDF format. The PDB database (https://www.rcsb.org/) was used to download the 3D structures of protein macromolecules in the PDB format. PyMoL 3.8.5 was used to remove water and ligands from the protein macromolecules. AutoDockTools 1.5.6 was used to convert macromolecules and compounds from the PDB to the PDBQT format, and the docking box position of protein macromolecular ligands was determined. AutoDock vina 1.1.2 script was used to dock protein macromolecules and compounds in sequence, obtain docking results, and draw molecular docking patterns (Zhou W. et al., 2021).

## 3 Results

### 3.1 Determination of the 7 phenolic acids in RPS and SPS

**FIGURE 3:**
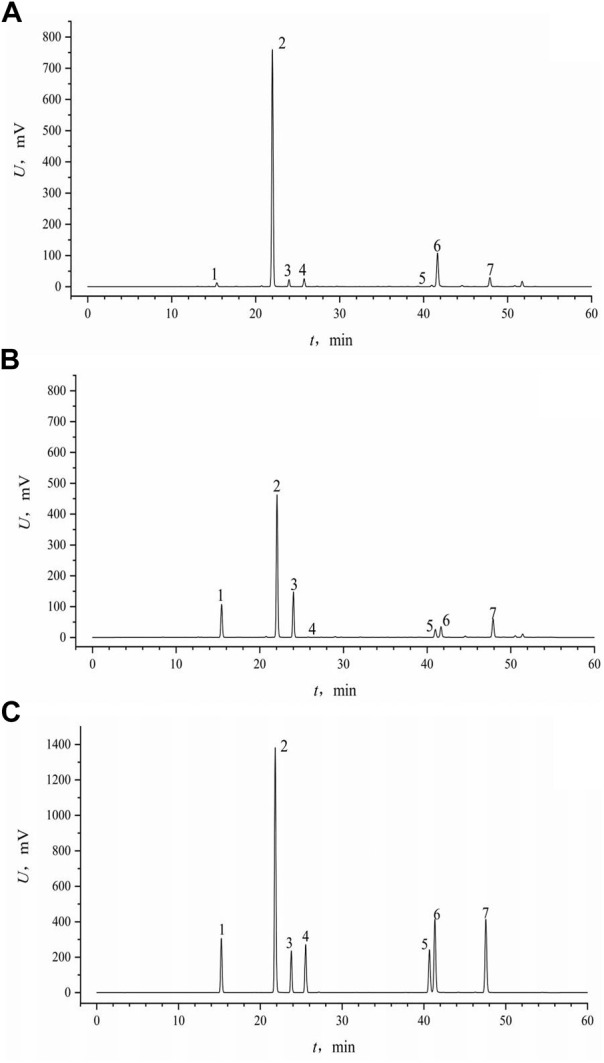
HPLC chromatograms of the raw sample **(A)**, processed sample **(B)**, and mixed standards **(C)** at 325 nm. 1.5-CQA; 2.3-CQA; 3.4-CQA; 4. CA; 5.3,4-DiCQA; 6.3,5-DiCQA; 7.4,5-DiCQA.

**FIGURE 4:**
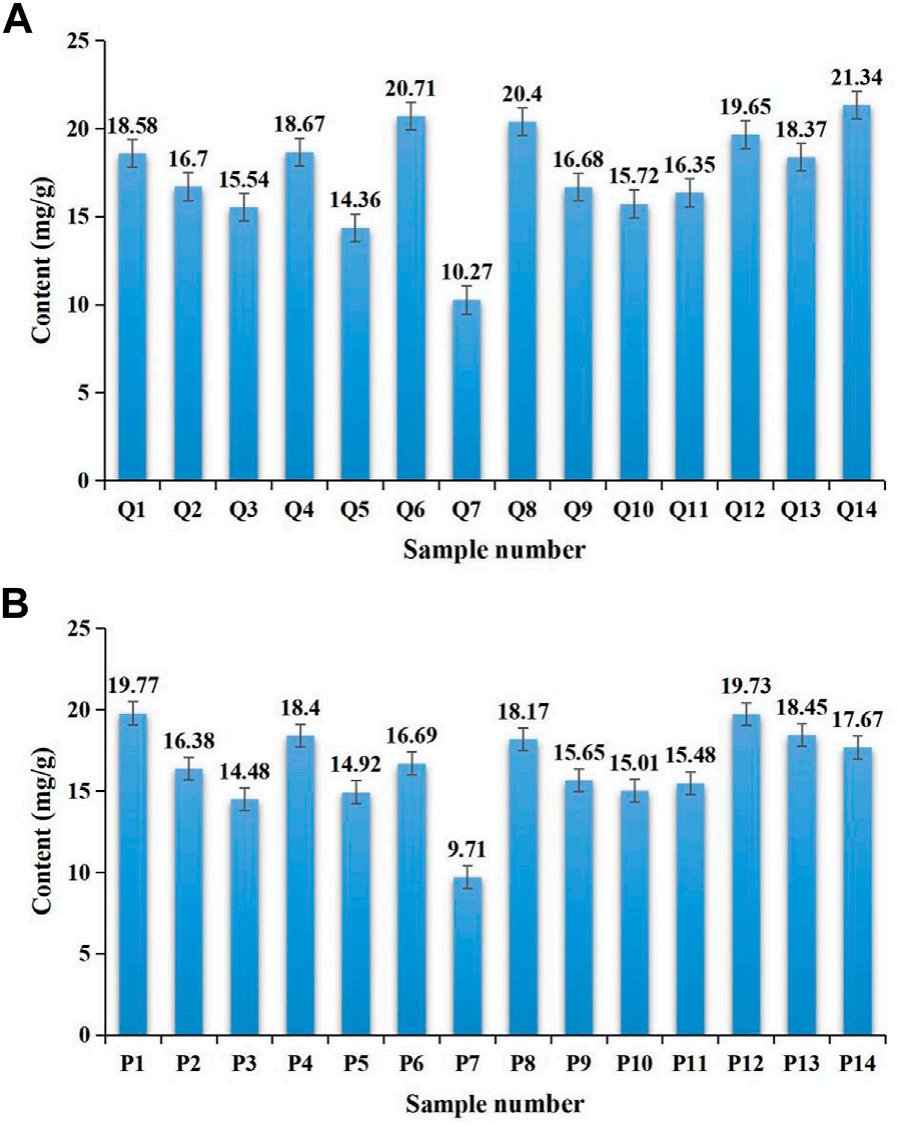
Total content of phenolic acids in the 14 batches of RPS **(A)** and SPS **(B)** samples.

An HPLC method was developed to determine the changes in the phenolic acids in PS during stir-frying (Figure 3). The total chemical content of each batch of PS changed significantly during the stir-fry processing. The level of each compound was analyzed. The levels of 3-CQA, CA, 3,5-DiCQA, and other compounds in most batches of PS were found to decrease after processing, whereas those of 5-CQA, 4-CQA, 3,4-DiCQA, and 4,5-DiCQA increased (Table 2). The total content of phenolic acids in the different batches of PS varied significantly (Figure 4). 3-CQA level was much higher than that of the other compounds, followed by 3,5-DiCQA level. Except for the sample procured from Jiangsu, 3-CQA content in other batches of PS was >1.07% and 3,5-DiCQA content was >0.13%; samples obtained from the Zhejiang, Sichuan, and Henan provinces were found to contain more phenolic acids in PS, with an average content of 2.13%, 1.90%, and 1.85%, respectively.

**TABLE 2:** Contents of the 7 phenolic acids in 14 batches of RPS and SPS samples.

Batch no.	5-CQA (mg/g)	3-CQA (mg/g)	4-CQA (mg/g)	CA (mg/g)	3,5-DiCQA (mg/g)	3,4-DiCQA (mg/g)	4,5-DiCQA (mg/g)	Total (mg/g)
Q1	0.35±0.01	13.66±0.01	0.86±0.01	0.49±0.00	2.05±0.02	0.33±0.00	0.84±0.01	18.58
P1	0.70±0.02	13.90±0.06	1.62±0.04	0.14±0.00	1.41±0.06	0.51±0.00	1.49±0.03	19.77
Q2	0.21±0.00	12.89±0.17	0.54±0.00	0.29±0.00	2.03±0.02	0.21±0.00	0.53±0.02	16.70
P2	0.45±0.03	12.13±0.19	1.10±0.03	0.23±0.01	1.21±0.06	0.31±0.01	0.95±0.02	16.38
Q3	0.19±0.00	11.78±0.11	0.49±0.00	0.32±0.01	2.06±0.02	0.21±0.00	0.49±0.02	15.54
P3	0.50±0.03	10.15±0.06	1.16±0.06	0.19±0.00	1.10±0.08	0.35±0.02	1.03±0.07	14.48
Q4	0.47±0.00	12.98±0.12	0.95±0.00	0.76±0.01	2.08±0.00	0.36±0.00	1.07±0.02	18.67
P4	0.65±0.00	12.63±0.03	1.44±0.00	0.33±0.01	1.43±0.01	0.47±0.00	1.45±0.02	18.40
Q5	0.30±0.00	10.75±0.27	0.64±0.02	0.61±0.01	1.31±0.03	0.21±0.00	0.54±0.02	14.36
P5	0.47±0.00	10.95±0.55	1.08±0.01	0.43±0.03	0.94±0.08	0.27±0.01	0.78±0.02	14.92
Q6	0.48±0.01	15.10±0.29	1.03±0.01	0.50±0.01	2.12±0.04	0.37±0.00	1.11±0.01	20.71
P6	0.65±0.03	11.42±0.73	1.39±0.07	0.25±0.02	1.20±0.11	0.45±0.02	1.33±0.08	16.69
Q7	0.44±0.01	6.22±0.08	0.82±0.00	0.78±0.01	0.91±0.02	0.36±0.00	0.74±0.01	10.27
P7	0.49±0.02	5.83±0.14	0.94±0.01	0.42±0.05	0.78±0.05	0.40±0.00	0.85±0.01	9.71
Q8	0.48±0.01	14.63±0.02	1.01±0.00	0.78±0.00	2.09±0.06	0.34±0.00	1.07±0.02	20.40
P8	0.63±0.08	12.70±0.25	1.38±0.17	0.35±0.08	1.33±0.01	0.43±0.05	1.35±0.13	18.17
Q9	0.22±0.01	12.76±0.11	0.60±0.00	0.25±0.00	2.03±0.05	0.22±0.00	0.60±0.02	16.68
P9	0.46±0.00	11.19±0.17	1.10±0.01	0.20±0.00	1.25±0.07	0.35±0.01	1.10±0.06	15.65
Q10	0.43±0.01	10.83±0.11	0.85±0.00	0.68±0.00	1.70±0.05	0.32±0.00	0.91±0.02	15.72
P10	0.61±0.04	9.98±0.44	1.31±0.06	0.26±0.02	1.15±0.06	0.44±0.02	1.26±0.07	15.01
Q11	0.18±0.00	1.240±0.08	0.54±0.01	0.21±0.00	2.30±0.07	0.22±0.00	0.50±0.01	16.35
P11	0.61±0.03	1.62±0.09	1.35±0.07	0.15±0.00	1.14±0.08	0.41±0.03	1.20±0.11	15.48
Q12	0.56±0.01	13.69±0.20	1.15±0.03	0.73±0.01	1.49±0.01	0.77±0.02	1.17±0.01	19.65
P12	0.88±0.00	13.20±0.43	1.88±0.01	0.24±0.02	1.18±0.09	0.89±0.02	1.46±0.07	19.73
Q13	0.54±0.01	12.47±0.03	1.21±0.02	0.36±0.00	1.81±0.04	0.94±0.00	1.04±0.02	18.37
P13	0.91±0.04	11.65±0.03	1.91±0.07	0.12±0.00	1.25±0.01	1.10±0.03	1.51±0.01	18.45
Q14	0.23±0.01	17.01±0.00	0.75±0.01	0.12±0.00	2.22±0.05	0.29±0.00	0.72±0.00	21.34
P14	0.64±0.04	12.87±0.03	1.53±0.05	0.06±0.00	0.96±0.02	0.42±0.01	1.19±0.02	17.67

### 3.2 Dynamic changes changes of the 7 phenolic acids during the stir-frying process of PS

**FIGURE 5:**
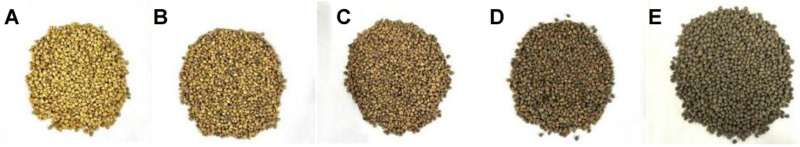
Raw PS sample and various stir-fried PS samples. **(A)**: raw sample; **(B)** and **(C)**: samples fried at 120°C for 3 min and 13 min; **(D)** and **(E)**: samples fried at 150°C for 3 min and 13 min.

The levels of three compounds, 3-CQA, CA, and 3,5-DiCQA decreased after processing, whereas those of the other four compounds increased significantly after processing. However, as only one processing temperature was used and it was for a short duration, the dynamic change rule of phenolic acid composition during the processing of PS cannot be fully revealed. Therefore, in this study, two processing temperatures were selected and the stir-frying time was extended to explore the change rule and trends of the 7 phenolic acids during stir-frying. The stir-frying temperatures were set at 120°C and 150°C, respectively, and the frying time was divided into 3, 5, 7, 9, 11, and 13 min. Figure 5 presents the images of the representative fried samples.

**FIGURE 6:**
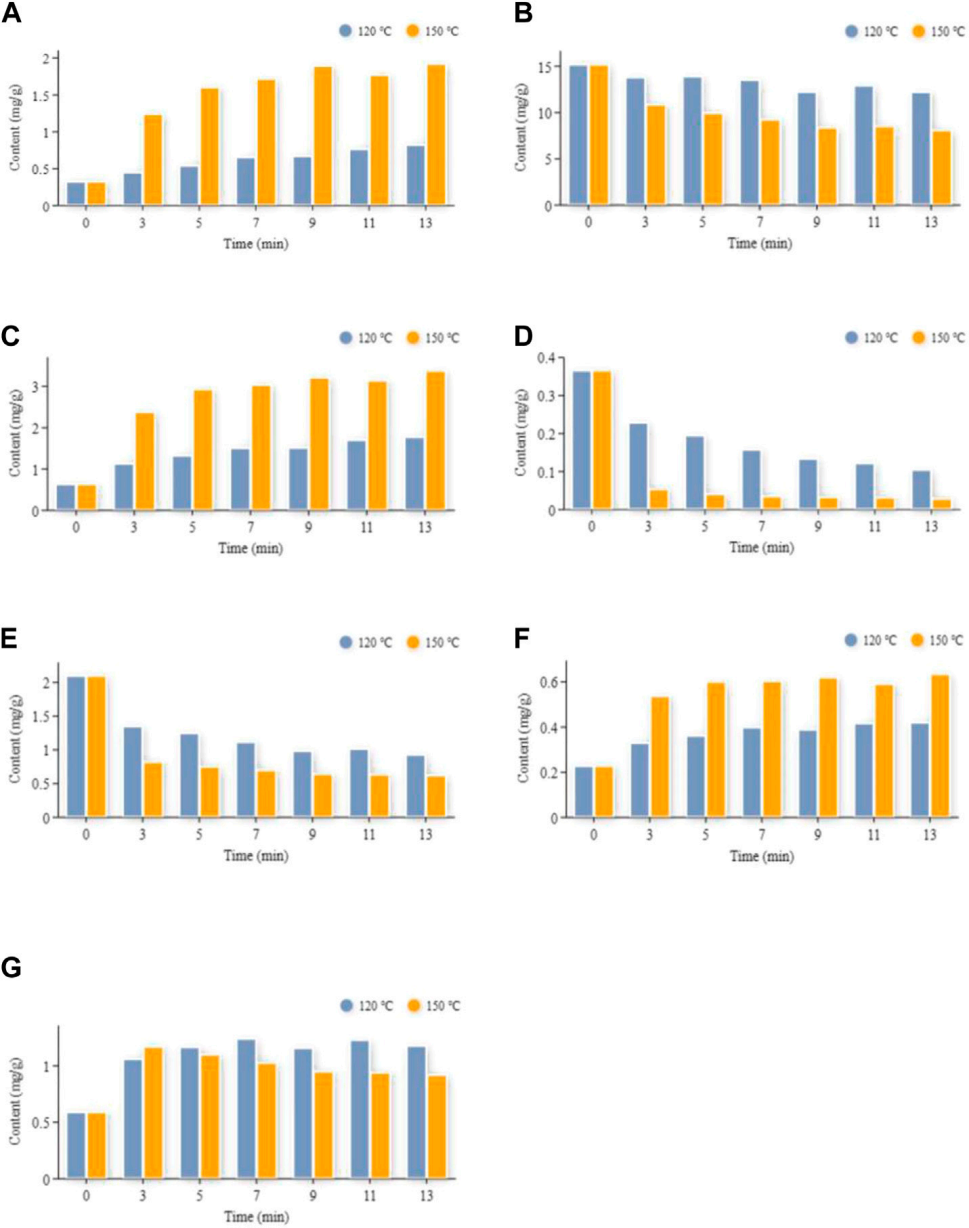
Change trend of the 7 phenolic acids in PS during the stir-frying process. **(A)**: 5-CQA; **(B)**: 3-CQA; **(C)**: 4-CQA; **(D)**: CA; **(E)**: 3,5-DiCQA; **(F)**: 3,4-DiCQA; **(G)**: 4,5-DiCQA.

**FIGURE 7:**
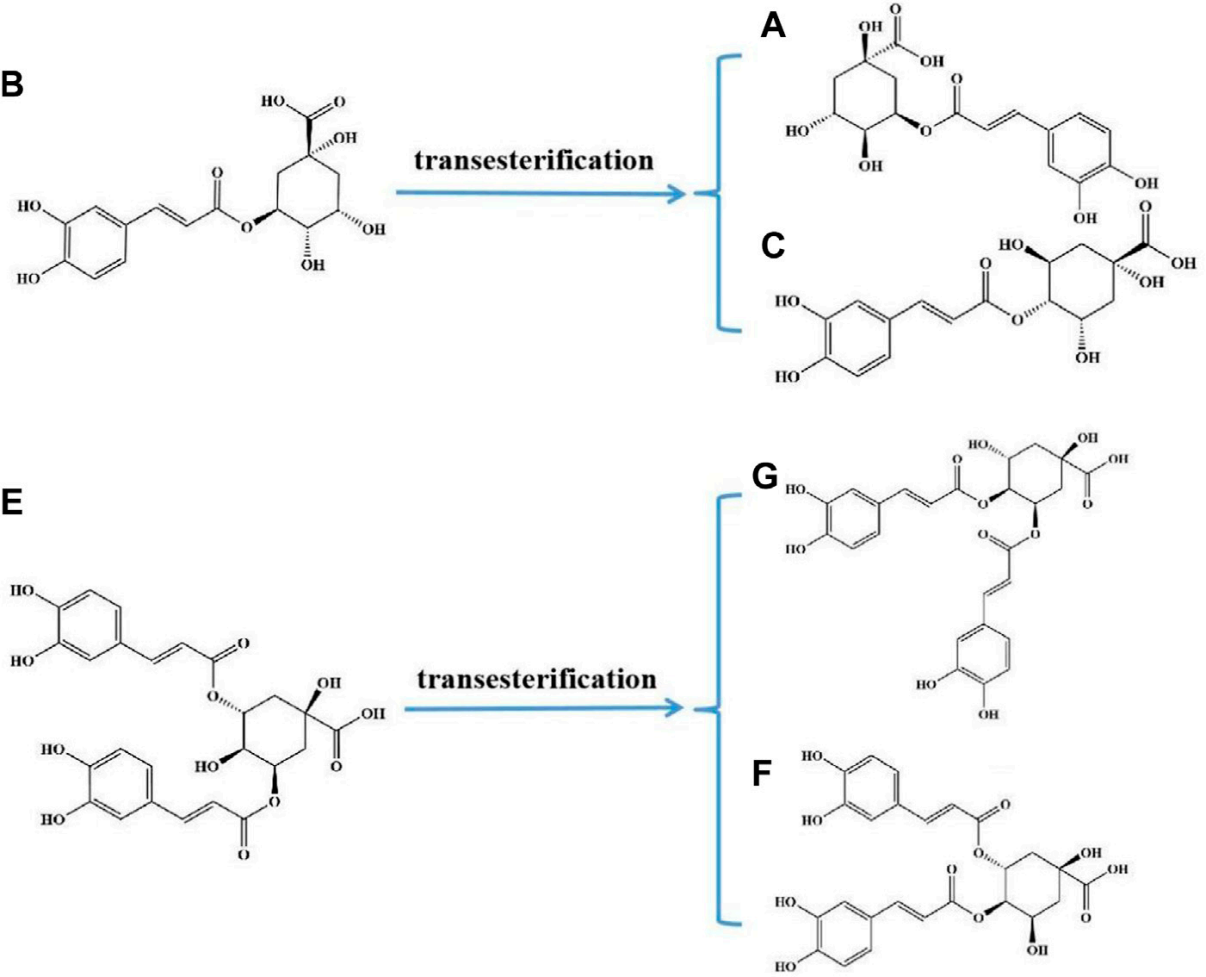
Possible chemical changes in the phenolic acids of PS during the stir-frying process. **(A)**: 5-CQA; **(B)**: 3-CQA; **(C)**: 4-CQA; **(D)**: CA; **(E)**: 3,5-DiCQA; **(F)**: 3,4-DiCQA; **(G)**: 4,5-DiCQA.

The % content of the 7 phenolic acids in the processed products (Tables 3, 4) and the trends in change of each compound during stir-frying are depicted in Figure 6. When heated for 0–13 min at 120 °C, the contents of 5-CQA, 4-CQA, 3,4-DiCQA, and 4,5-DiCQA showed an overall increasing trend, whereas those of 3-CQA, CA, and 3,5-DiCQA showed a generally decreasing trend. When heated for 0–13 min at 150 °C, the contents of 5-CQA, 4-CQA, 3,4-DiCQA, and other compounds increased significantly compared with that in the raw product and during the stir-frying process at 120 °C; the contents of 3-CQA, CA, and 3,5-DiCQA decreased significantly compared with that in the raw product and during stir-fry processing at 120°C. The content of 4,5-DiCQA was the highest when stir-fried for 3 min and decreased with an increase in stir-frying time. As chlorogenic acid, neochlorogenic acid, and cryptochlorogenic acid, as well as isochlorogenic acids A, B, and C are isomers, we speculated that an ester-exchange reaction occurred during the stir-frying process, leading to significant changes in the contents of these six components (Figure 7).

**TABLE 3:** Content changes of the main compounds in PS during the stir-frying process (120°C).

Processing time (min)	Content (mg/g)						
	5-CQA	3-CQA	4-CQA	CA	3,5-DiCQA	3,4-DiCQA	4,5-DiCQA
0	0.316	15.111	0.619	0.364	2.089	0.225	0.584
3	0.441	13.734	1.114	0.227	1.340	0.327	1.055
5	0.532	13.830	1.309	0.193	1.239	0.359	1.160
7	0.647	13.456	1.494	0.156	1.107	0.396	1.234
9	0.662	12.163	1.499	0.132	0.975	0.386	1.152
11	0.757	12.853	1.687	0.120	1.006	0.414	1.224
13	0.814	12.148	1.760	0.103	0.919	0.417	1.172

**TABLE 4:** Content changes of the main compounds in PS during the stir-frying process (150 °C).

Processing time (min)	Content (mg/g)						
	5-CQA	3-CQA	4-CQA	CA	3,5-DiCQA	3,4-DiCQA	4,5-DiCQA
0	0.316	15.111	0.619	0.364	2.089	0.225	0.584
3	1.234	10.787	2.365	0.052	0.811	0.534	1.163
5	1.595	9.881	2.916	0.039	0.742	0.598	1.094
7	1.709	9.180	3.022	0.033	0.689	0.601	1.021
9	1.885	8.311	3.200	0.031	0.634	0.617	0.944
11	1.762	8.461	3.125	0.030	0.625	0.588	0.935
13	1.913	8.027	3.362	0.027	0.612	0.632	0.914

### 3.3 Network analysis

#### 3.3.1 Prediction of candidate targets and construction of the “compound-target” network

**FIGURE 8:**
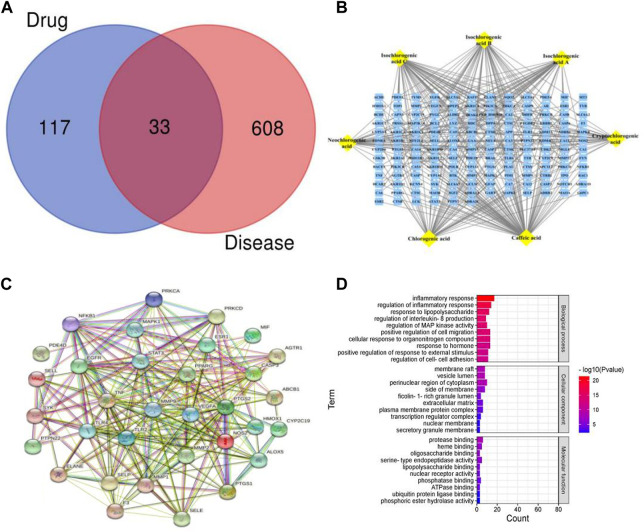
Network analysis of PS in treating nephritis. **(A)** Venn diagram of the 7 phenolic acids and targets of nephritis; **(B)** Compound-target network for the 7 phenolic acids; **(C)** PPI interaction network diagram; **(D)** Top 30 enriched GO terms for the biological processes of the potential targets.

Nephritis-related genes were obtained by searching the keywords “Nephritis” and “Glomerulonephritis”; 129 disease targets were obtained from the OMIM database, 9 from the TTD database, and 575 from the GeneCards database after the median screening. A total of 641 nephritis-related disease targets were finally obtained after summarizing, deduplication, and union. A Venn diagram was drawn between the targets of the 7 phenolic acids and the targets of nephritis-related diseases, and a total of 33 crossover targets were obtained (Figure 8A). Using SwissTargetPrediction, the species was set to human to obtain the targets of the 7 phenolic acids. They were combined with the targets screened using the HIT 2.0 and HERB Materia Medica databases to remove duplicates and to get new targets, respectively. After summarizing and duplicating the targets of the 7 phenolic acids, 150 compound targets were obtained, which were imported into Cytoscape 3.7.1 together with the compound information to construct a compound–target network diagram (Figure 8B). The network graph has 157 nodes and 324 edges. The yellow diamonds represent the phenolic acids, blue squares represent the relevant targets of the compounds, and edges represent the interactions between the compounds and targets.

#### 3.3.2 Protein-protein interaction network core target screening

The 33 compounds and disease intersection targets that were screened out were imported into the STRING database to generate a protein-protein interaction network map. Nodes represent the target proteins and edges represent the functional correlations between proteins. There are 33 nodes and 255 edges in total, with an average node degree of 15.5 and an average local clustering coefficient of 0.755 (Figure 8C). The interaction relationship between the core target proteins was made more intuitive by importing the TSV file into Cytoscape 3.7.1 for visual analysis. The top 10 were selected as the core targets according to the ranking of degree value, followed by tumor necrosis factor (TNF), vascular endothelial growth factor A (VEGFA), prostaglandin G/H synthase 2 (PTGS2), matrix metalloproteinase 9 (MMP9), toll-like receptor 4 (TLR4), signal transducer and activator of transcription 3 (STAT3), human epidermal growth factor receptor (EGFR), caspase 3 (CASP3), toll-like receptor 2 (TLR2), and peroxisome proliferator–activated receptor gamma (PPARG). It can be seen that the targets TNF, VEGFA, and PTGS2 have larger patterns and darker colors than the other targets. Thus, we speculated that these three targets could be the key targets for the treatment of nephritis by PS.

#### 3.3.3 GO biological process and KEGG pathway enrichment analysis

The intersection targets were imported into the Metascape database for GO enrichment analysis, and entries with *p* < 0.01 were screened out; there were 510 biological processes, 57 molecular functions, and 38 cellular components. Based on the *p*-value, the top 10 items with representative biological processes, molecular functions, and cellular compounds were screened out (Tables 5–7), and GO enrichment bar charts were constructed (Figure 8D). Biological processes mainly involved the inflammatory response, regulation of the inflammatory response, lipopolysaccharide response, regulation of interleukin-8 production, and regulation of MAP kinase activity among others. Molecular functions mainly involved protease binding, heme binding, oligosaccharide binding, and lipopolysaccharide binding among others; cell compounds mainly involved membrane rafts, vesicle lumen, cytoplasmic perinuclear region, membrane side, and ficoli-1-rich granule lumen. GO biological process data showed that the 7 phenolic acids may be involved in the regulation of these items to possibly achieve a therapeutic effect in treating nephritis. KEGG pathway enrichment analysis was performed on the intersecting targets by using the Metascape database with *p* < 0.01 and an enrichment factor of >1.5. A total of 126 signaling pathways of the 7 phenolic acids were screened and found to interfere with nephritis. The top 15 pathways were filtered from the smallest to the largest *p*-value (Table 8). Nephritis targets are mainly enriched in the AGE-RAGE, HIF-1, IL-17, and TNF signaling pathways during diabetic complications.

**TABLE 5:** Results from GO biological process.

Numbering	English name	Number of targets	*p*-Value
GO:0006954	inflammatory response	17	4.16869E-22
GO:0050727	inflammatory response	17	4.16869E-22
GO:0032496	regulation of inflammatory response	14	2.13796E-18
GO:0032677	response to lipopolysaccharide	12	4.36516E-16
GO:0043405	regulation of interleukin-8 production	9	1.44544E-15
GO:0030335	regulation of MAP kinase activity	10	4.2658E-15
GO:0071417	positive regulation of cell migration	13	1.09648E-14
GO:0009725	cellular response to organonitrogen compound	13	1.44544E-14
GO:0032103	response to hormones	13	3.89045E-13
GO:0022407	positive regulation of response to external stimulus	11	8.51138E-13

**TABLE 6:** Results from GO molecular function.

Numbering	English name	Number of targets	*p*-Value
GO:0002020	protease binding	6	7.4131E-09
GO:0020037	heme binding	5	4.0738E-07
GO:0070492	oligosaccharide binding	3	5.37032E-07
GO:0004252	serine-type endopeptidase activity	5	1.25893E-06
GO:0001530	lipopolysaccharide binding	3	6.91831E-06
GO:0004879	nuclear receptor activity	3	2.51189E-05
GO:0019902	phosphatase binding	4	5.7544E-05
GO:0051117	ATPase binding	3	0.0001
GO:0031625	ubiquitin protein ligase binding	3	0.004168694
GO:0042578	phosphoric ester hydrolase activity	3	0.007413102

**TABLE 7:** Results from GO cell component.

Numbering	English name	Number of targets	*p*-Value
GO:0045121	membrane raft	8	1.86209E-09
GO:0031983	vesicle lumen	8	1.90546E-09
GO:0048471	perinuclear region of cytoplasm	10	3.0903E-09
GO:0098552	side of membrane	8	4.57088E-07
GO:1904813	ficolin-1-rich granule lumen	4	0.00001
GO:0031012	extracellular matrix	6	3.0903E-05
GO:0098797	plasma membrane protein complex	6	0.000102329
GO:0005667	transcription regulator complex	4	0.001819701
GO:0031965	nuclear membrane	3	0.004265795
GO:0030667	secretory granule membrane	3	0.004786301

**TABLE 8:** Results from KEGG pathway enrichment analysis.

Numbering	English name	Number of targets	*p*-Value
HSA04933	AGE-RAGE signaling pathway in diabetic complications	12	2.95121E-22
HSA05417	lipid and atherosclerosis	13	4.16869E-20
HSA05200	pathways in cancer	16	5.88844E-20
HSA05171	coronavirus disease - COVID-19	12	9.77237E-18
HSA04066	HIF-1 signaling pathway	9	2.63027E-15
HSA04926	relaxin signaling pathway	9	1.25893E-14
HSA05161	hepatitis B	9	1.02329E-13
HSA04657	IL-17 signaling pathway	7	8.91251E-12
HSA04668	TNF signaling pathway	7	3.16228E-11
HSA04726	serotonergic synapse	7	3.80189E-11
HSA04625	C-type lectin receptor signaling pathway	6	1.47911E-09
HSA04151	PI3K-Akt signaling pathway	8	3.54813E-09
HSA04915	estrogen signaling pathway	6	8.12831E-09
HSA05321	inflammatory bowel disease	5	8.91251E-09
HSA04620	toll-like receptor signaling pathway	5	9.54993E-08

#### 3.3.4 Analysis of the “compound-target-signaling pathway” network

**FIGURE 9:**
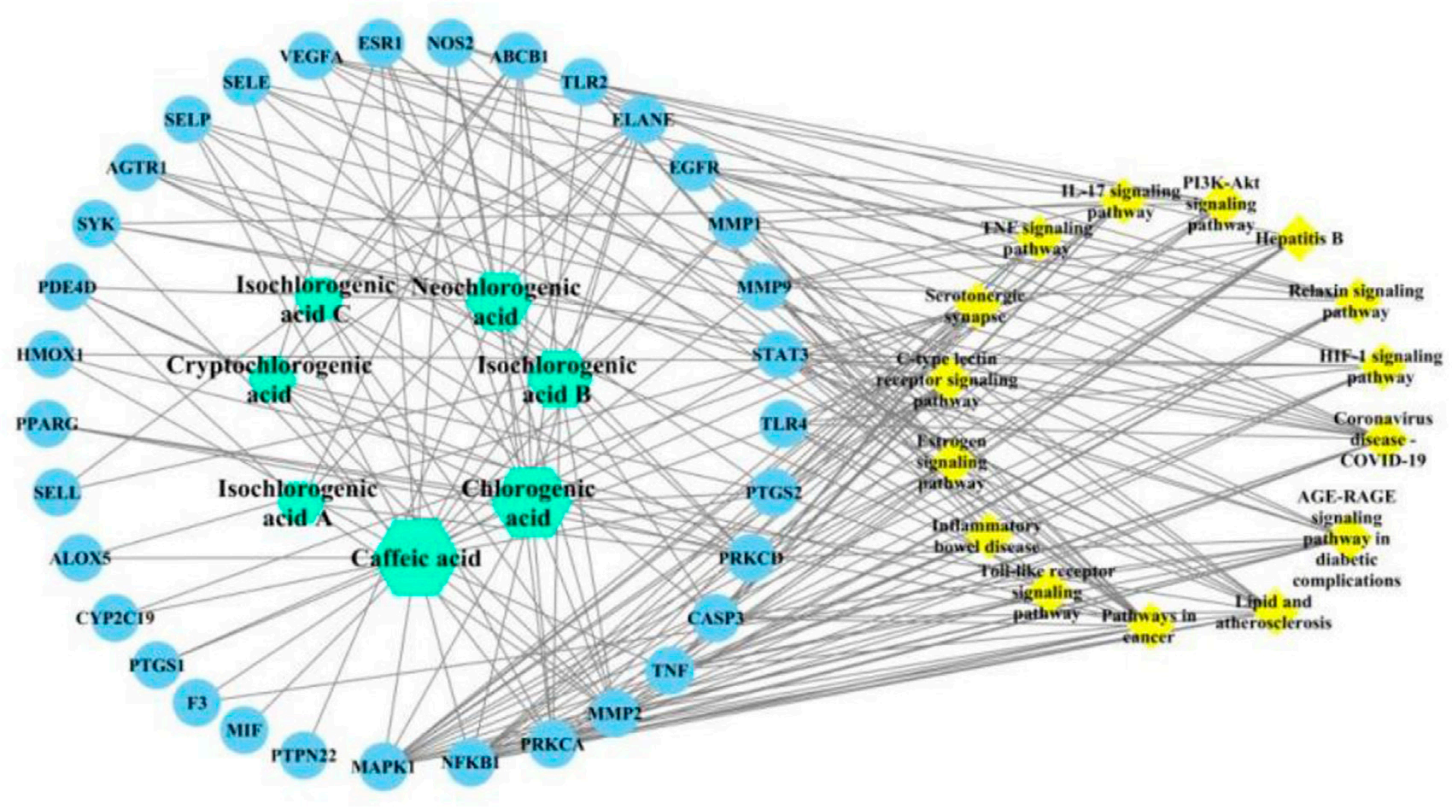
Target-pathway network of the 7 phenolic acids in treating nephritis.

The 7 phenolic acids, intersection targets, and results from the KEGG pathway were integrated and processed using Cytoscape 3.7.1 to construct a component-target-signaling pathway network diagram and for visual analysis (Figure 9). There are 55 nodes and 209 edges in the graph; the blue circles represent targets, green hexagons represent the 7 phenolic acids, and yellow diamonds represent the KEGG pathway. It can be seen that CA, 3-CQA, 3,4-DiCQA, and 5-CQA have larger nodes than the other 3 compounds, indicating that the 4 compounds have more nephritis targets and may be the key compounds in treating nephritis. The key targets such as TNF, VEGFA, PTGS2, MMP9, and MAPK1 are directly or indirectly related to the 7 phenolic acids and multiple nephritis-related pathways. Therefore, the treatment of nephritis by these 7 phenolic acids is *via* multiple targets and pathways.

### 3.4 Molecular docking

Molecular docking was used to validate the results of the above network analysis, screen the network of the 7 phenolic acids with high degree values, and dock them with the targets of action on nephritis. Based on the analysis (Table 9), TNF, VEGFA, PTGS2, MMP9, and MAPK1, which were ranked the highest in degree value, were selected as receptor proteins. It is believed that the prerequisite for the binding of small molecules to protein macromolecules is that the binding energy should be <0; moreover, the lower the binding energy value, the greater the binding possibility. The pairing result with the highest binding affinity was CA and TNF (–3.9 kcal/mol), and the pairing results with the second- and third-highest binding affinities were in the case of 3,5-DiCQA and PTGS2 (–5.4 kcal/mol) and 3-CQA, 5-CQA, 4-CQA, and MAPK1 (–7.0 kcal/mol), which suggested that TNF, PTGS2, and MAPK1 may be the key targets.

**TABLE 9:** Results from molecular docking.

Compounds	Binding energy (kCal/mol)				
	TNF	VEGFA	PTGS2	MMP9	MAPK1
CA	−3.9	−6.4	−6.6	−7.6	−5.7
3-CQA	−4.4	−8.1	−7.5	−9.6	−7.0
3,4-DiCQA	−5.4	−8.7	−6.4	−10.0	−7.1
5-CQA	−4.2	−8.1	−7.6	−9.0	−7.0
4,5-DiCQA	−4.0	−8.5	−5.5	−10.0	−7.7
4-CQA	−4.4	−7.8	−7.1	−9.4	−7.0
3,5-DiCQA	−4.8	−8.4	−5.4	−9.1	−7.3

**FIGURE 10:**
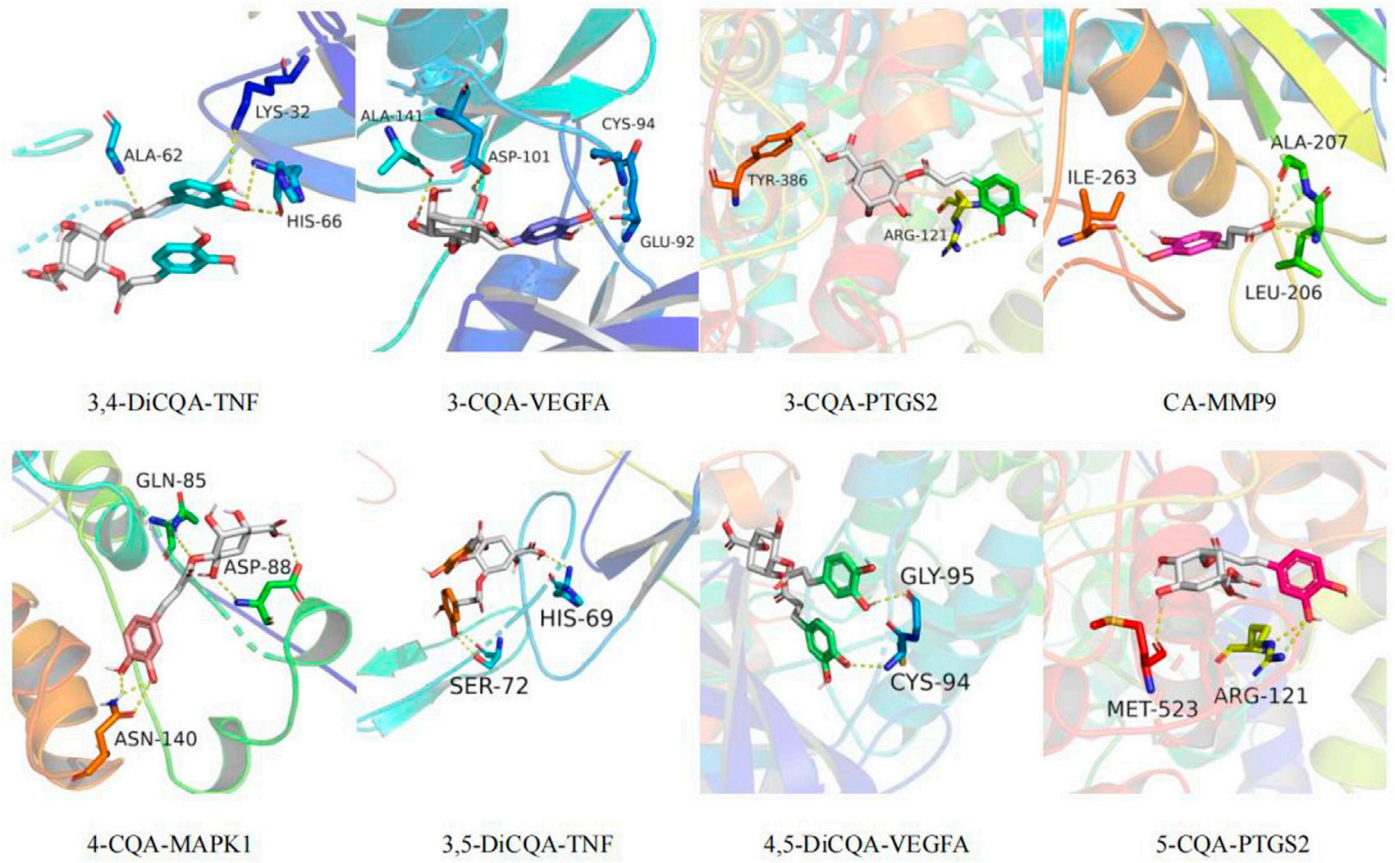
Docking mode of the 7 phenolic acids with their key targets.

The pattern of molecular docking results between some of these components and the target is shown in Figure 10. Molecular docking results showed that the 7 phenolic acids could bind well to the nephritis targets, among which CA, 3,5-DiCQA, 3-CQA, 5-CQA, and 4-CQA had good binding affinity with each target. The docking results between the targets of VEGFA and MMP9 and each component were also good, which may play an essential role in preventing as well as treating nephritis (Liu C. D. et al., 2023; Sanaya et al., 2023). Molecular docking also revealed that 3-CQA had good binding ability with TNF. These results were similar to the findings reported by Liu et al., wherein 3-CQA had good binding ability with TNF (Liu J. H. et al., 2023; Xu et al., 2023). Both theories indicated that 3-CQA exhibited the potential to prevent and treat nephritis. These results suggested that the 7 phenolic acids play an important role in the treatment of nephritis and that these targets are also important target proteins in treating nephritis.

## 4 Discussion

Phenolic acids have good antinephritic effects (Ki et al., 2012); network analysis and molecular docking were performed to evaluate the role of the 7 phenolic acids in PS in treating nephritis. The results showed that CA, 3,5-DiCQA, 3-CQA, 5-CQA, and 4-CQA had good binding affinities with key targets and may play a more significant role in the treatment of nephritis. Previous studies have shown that CA can prevent the development of diabetic nephropathy by downregulating miR-636 expression (Salem et al., 2019). 3-CQA alleviates renal ischemia-reperfusion injury by reducing inflammation, reducing myofibroblast expansion, and inducing epithelial cell proliferation (Arfian et al., 2019). The protective effect of 3-CQA against renal injury may be attributed to its antioxidant and anti-inflammatory activities (Feng et al., 2016). 3-CQA, 3,4-DiCQA, and 4,5-DiCQA, identified as anti-inflammatory quality markers using network analysis, were subjected to ultra-high performance liquid chromatography and biological activity verification (Zhou Y. F. et al., 2021). With an increase in 5-CQA concentration, the inhibition rate of the inflammatory factor IL-6 increased correspondingly (Gao et al., 2020). *Acanthopanax senticosus* extract contains 3-CQA, 3,5-DiCQA, and 4,5-DiCQA, which have strong anti-inflammatory effects (Chen et al., 2021). 4-CQA exerts anti-inflammatory effects by reducing oxidative stress during the inflammatory response (Zhao et al., 2020). Therefore, previous studies have proved that these ingredients can act on relevant targets and pathways, and then exert anti-inflammatory, antioxidant, and other pharmacological effects, further proving the reliability of the results of this study (Zhang, 2021).

The protein interaction analysis network showed that TNF, VEGFA, PTGS2, MMP9, MAPK1, and other proteins were the key targets of the 7 phenolic acids in the treatment of nephritis. TNF-α from the TNF family is involved in the inflammatory response of chronic nephritis, and it is used as a detection index in the acute infectious phase of chronic nephritis (Wu et al., 2010; Yuan et al., 2018). Activation of the mesangial cells in mesangial proliferative glomerulonephritis significantly increases VEGFA expression. PTGS2, also known as cyclooxygenase 2, induces prostaglandin production and plays a key role in regulating inflammatory responses (Jason et al., 2015). High MMP9 expression alters the blood–brain barrier permeability, increasing the possibility of viral infections and susceptibility to inflammatory factors and inflammatory cell infiltration (Qiao et al., 2022). It has been reported that MAPK1 expression significantly increases in model groups of nephritis.

Findings from KEGG enrichment pathway analysis and bubble plots revealed that glomerulonephritis targets were mainly enriched in AGE-RAGE, HIF-1, IL-17, and TNF signaling pathways in diabetic complications. Inhibition of the inflammatory channels activated by the AGE-RAGE signaling pathway can reduce the inflammatory response in diabetic nephropathy, thereby protecting the kidneys (Malik and Kumar, 2022). HIF primarily responds to and mediates the hypoxia response, contributing to the development of renal fibrosis and inflammation during chronic kidney disease (Liu et al., 2017). The proinflammatory cytokine IL-17 is upregulated in endothelial cells during the pathogenesis of acute anti-thy1 glomerulonephritis (Loof et al., 2016). TNF is a cytokine that mediates inflammatory kidney disease, and the exclusive expression of transmembrane TNF exacerbates acute glomerulonephritis (Müller et al., 2019). Based on the above results, it could be concluded that the 7 phenolic acids in PS exerted their effects in treating nephritis through various pathways such as the cellular metabolic pathway, apoptotic pathway, and inflammatory pathway. In addition, many other signaling pathways have been shown; however, their mechanisms of action need to be further explored.

## 5 Conclusion

In this study, the content changes in the 7 phenolic acids in PS during the stir-frying process were determined using HPLC. The 7 phenolic acids of PS could effectively treat nephritis through multiple pathways and multiple targets. The accuracy of the outcomes was further verified using subsequent molecular docking. The results showed that the 7 phenolic acids of PS could regulate the proliferation, metastasis, differentiation, senescence, apoptosis, and other biological processes of inflammatory cells by regulating the expression of related genes in nephritic cells, reflecting the antinephritic effect of PS. These findings illustrate the complexity of the pathological mechanisms of nephritis and the diversity of pharmacological activities of the phenolic acids in PS. However, our research results need to be further corroborated using animal experiments and other relevant studies to ensure their reliability. Overall, our study provides a novel basis for further exploration and subsequent experimental verification of PS in the treatment of nephritis.

## Data Availability

The original contributions presented in the study are included in the article/supplementary material, further inquiries can be directed to the corresponding authors.
